# Intrafamilial Phenotypic Variability of the *FGFR1* p.Cys277Tyr Variant: A Case Report and Review of the Literature

**DOI:** 10.3390/genes16050495

**Published:** 2025-04-26

**Authors:** Anna Szoszkiewicz, Anna Sowińska-Seidler, Karolina Gruca-Stryjak, Aleksander Jamsheer

**Affiliations:** 1 Doctoral School, Department of Medical Genetics, Poznan University of Medical Sciences, Rokietnicka 8, 60-806 Poznan, Poland; 2 Department of Medical Genetics, Poznan University of Medical Sciences, Rokietnicka 8, 60-806 Poznan, Poland; seidler@ump.edu.pl; 3 Perinatology Department, Poznan University of Medical Sciences, Polna 33, 60-535 Poznan, Poland; karolagruca@poczta.onet.pl; 4 Diagnostyka GENESIS, Dąbrowskiego 77A, 60-529 Poznan, Poland

**Keywords:** fibroblast growth factor receptor, cleft lip, congenital limb defect, ectrodactyly, split-hand/foot malformation, hyposmia, next-generation sequencing, genotype–phenotype correlation

## Abstract

Background: Split-hand/foot malformation (SHFM) is a rare congenital limb anomaly defined by the absence or hypoplasia of the central rays of the autopod. SHFM occurs as an isolated entity or part of genetic syndromes with several causative copy-number variations or monogenic alterations known to be involved in the disease pathomechanism. On the other hand, cleft lip/palate (CL/P) usually results from polygenic and environmental factors, with the complex interplay of both leading to this malformation. Pathogenic variants in *FGFR1* have been linked to phenotypically distinct disorders, including Hartsfield syndrome, Kallmann syndrome, Jackson–Weiss syndrome, osteoglophonic dysplasia, and Pfeiffer syndrome. Although pathogenic variants in *FGFR1* can contribute to syndromic SHFM or CL/P, their role in isolated SHFM or CL remains poorly described in the literature. Methods: We conducted targeted next-generation sequencing (NGS) in the proband with SHFM, followed by segregation analysis in the family members. Results: In this study, we report an index patient presenting with isolated SHFM and his brother with CL and facial dysmorphism, as well as their father with isolated hyposmia. Targeted next-generation sequencing revealed a previously reported heterozygous missense pathogenic variant in *FGFR1* (c.830G>A; p.Cys277Tyr) in both affected siblings and their hyposmic father. Conclusions: This study expands the phenotypic spectrum associated with *FGFR1* pathogenic variants, emphasizing their involvement in non-syndromic SHFM and CL or isolated hyposmia. Our findings highlight the importance of considering *FGFR1* in the molecular diagnosis of isolated SHFM or orofacial clefting, point to the high intrafamilial variability of *FGFR1* pathogenic variants, and demonstrate the diagnostic value of targeted NGS in rare congenital malformations.

## 1. Introduction

Split-hand/foot malformation (SHFM), also known as ectrodactyly, is a rare congenital limb malformation with a prevalence of 1 per 18,000 live births, which is characterized by median clefts of the hands and/or feet, syndactyly, aplasia or hypoplasia of the phalanges, metacarpals, and metatarsals [1]. The disorder exhibits genetic and phenotypic heterogeneity and typically inherits as an autosomal dominant trait, showing in affected families highly variable expressivity. SHFM may occur as an isolated feature or as a part of a syndrome [2,3]. Thirteen chromosomal regions are associated with ectrodactyly, corresponding to the SHFM classification. For seven loci, the underlying genes have been identified, including *DLX5* and *DLX6* (SHFM1), *TP63* (SHFM4), *WNT10B* (SHFM6), *FGFR1* (HH2), *MAP3K20* (SFMMP), *UBA2* (ACCES), and *EPS15L1* (SHFM8). In addition, type 3 of split hand foot malformation with long bone deficiency (SHFLD3) is associated with duplications at 17p13.3 encompassing the *BHLHA9* gene, whereas SHFM3 is linked to duplications at 10q24.3 resulting in ectopic expression of several genes at the *LBX1/FGF8* locus [4,5]. The molecular mechanism and disease-associated genes remain elusive for the remaining types of the disorder. Most patients follow an autosomal dominant inheritance pattern with a relatively frequent incidence of incomplete penetrance.

An isolated cleft lip/palate (CL/P), a sporadic facial malformation, is polygenic in nature. Several known genetic and environmental risk factors responsible for an isolated form of CL/P have been identified, with insufficient periconceptional folic acid supplementation being the most frequently emphasized [6]. In addition, there are a number of chromosomal aberrations or monogenic syndromes with CL/P as one of the phenotypic features. For example, trisomy 13, 3q29 microdeletions, or *IRF6* pathogenic variants cause various syndromic forms of orofacial clefting [7,8].

Pathogenic variants in the *FGFR1* (fibroblast growth factor receptor 1) gene can cause broad phenotypic spectrum, including Hartsfield syndrome, Jackson–Weiss syndrome, Kallmann syndrome (KS) type 2, hypogonadotropic hypogonadism without hyposmia (HH), osteoglophonic dysplasia, Pfeiffer syndrome, and Trigonocephaly 1 via autosomal-dominant inheritance [9,10,11,12,13,14]. Furthermore, heterozygous loss-of-function pathogenic variants in *FGFR1* can cause isolated SHFM and congenital hypogonadotropic hypogonadism (CHH) with SHFM [15,16,17]. FGFR1 exhibits high expression across multiple tissues, including the cranial neural crest (CNC)-derived palate mesenchyme, the brain, and the skeletal system. The protein is critical during early limb development, particularly in autopod formation and digit positioning [18,19,20].

In this study, we examined a Polish family with a proband affected by SHFM, for whom we performed a targeted NGS screening of SHFM-related genes. Next, we analyzed his brother, who presented with a CL and minor digital abnormalities, and their hyposmic father. Finally, we performed a segregation analysis of all other unaffected family members to determine the inheritance pattern. This study provides a detailed genotype–phenotype correlation within the family carrying the variant in *FGFR1* (c.830G>A; p.Cys277Tyr) and places them within the context of previously documented *FGFR1*-related disorders.

## 2. Materials and Methods

We collected peripheral blood from the index and his relatives. Next, we extracted genomic DNA (gDNA) using the MagCore HF16 Automated Nucleic Acid Extractor (RBC Bioscience, New Taipei City, Taiwan) and quantified using Agilent 2200 TapeStation System (Agilent Technologies, Santa Clara, CA, USA) and Qubit fluorometer (Thermo Fisher Scientific, Waltham, MA, USA). Written informed consent was obtained from the patients and their legal guardians prior to genetic testing. This study was approved by the Institutional Review Board of the Poznan University of Medical Sciences Ethics Committee.

### 2.1. Next-Generation Sequencing (NGS) Analysis

We designed a gene panel using the IonAmpliSeq Designer v7.8.7 platform (Thermo Fisher Scientific, Waltham, MA, USA), targeting 25 genes associated with SHFM or candidates (Table S1). We selected genes for the SHFM panel based on a review of the medical literature and their documented association with SHFM in databases (OMIM and ClinVar). We used high-quality DNA at an input amount of 50 ng, with a DNA Integrity Number (DIN)8.9. The libraries were synthesized manually according to the manufacturer’s protocol (Ion AmpliSeq™ Library Kit 2.0, Thermo Fisher Scientific, Waltham, MA, USA). Targeted next-generation sequencing (NGS) was performed on the Ion Torrent S5 System following the Ion AmpliSeq Library Kit 2.0 protocol (Thermo Fisher Scientific, Waltham, MA, USA). The detailed procedure was described by Bukowska-Olech et al. (2020) [21]. Sequence data were processed using the Torrent Suite 5.20.8.0 pipeline software to analyze raw data, base calling, filter out low-quality reads, and map to the human genome (GRCh37/hg19). Variant selection was performed using the following filtering criteria: read depth ≥ 20, PHRED score ≥ 40, and variant frequency ≥ 0.15. We visualized read alignments using Integrative Genomics Viewer (Version 2.19.1, Broad Institute and the Regents of the University of California). Identified variants were cross-referenced with the Human Gene Mutation Database (HGMD) (https://www.hgmd.cf.ac.uk/ac/index.php) (accessed on 11 December 2024), ClinVar (https://www.ncbi.nlm.nih.gov/clinvar/) (accessed on 11 December 2024), dbSNP (https://www.ncbi.nlm.nih.gov/snp/) (accessed on 11 December 2024), and GnomAD browser (Genome Aggregation Database) (https://gnomad.broadinstitute.org/) (accessed on 11 December 2024). Pathogenicity was assessed using SIFT, CADD, REVEL, and other resources integrated into the VarSome Premium online tool v.12.9.0 (Kopanos et al., 2019) [22]. Variants were classified according to the American College of Medical Genetics and Genomics (ACMG) guidelines using the VarSome Premium platform v.12.9.0 [22].

### 2.2. Sanger Sequencing

Polymerase chain reaction (PCR) followed by Sanger sequencing was performed to confirm the selected variants in the patient and family members available for genetic testing. We designed primers using the Primer3 tool v. 0.4.0 (Table S2). We conducted PCR in a reaction mixture containing 5 μL of FailSafe™ PCR 2X PreMix J (Lucigen Epicentre), 2.9 μL of PCR-grade water, 0.5 μL of primers (10 μmol/L each), 0.1 μL of Taq DNA polymerase (GenScript), and 1 μL of gDNA. Amplified DNA was purified using standard protocols. PCR products were sequenced using dye-terminator chemistry (kit v.3, ABI 3130xl) and run on an Applied Biosystems PRISM 3700 DNA sequencer (Thermo Fisher Scientific, Waltham, MA, USA). The reaction conditions are available upon request.

## 3. Results

### 3.1. Clinical Report

**Figure 1:**
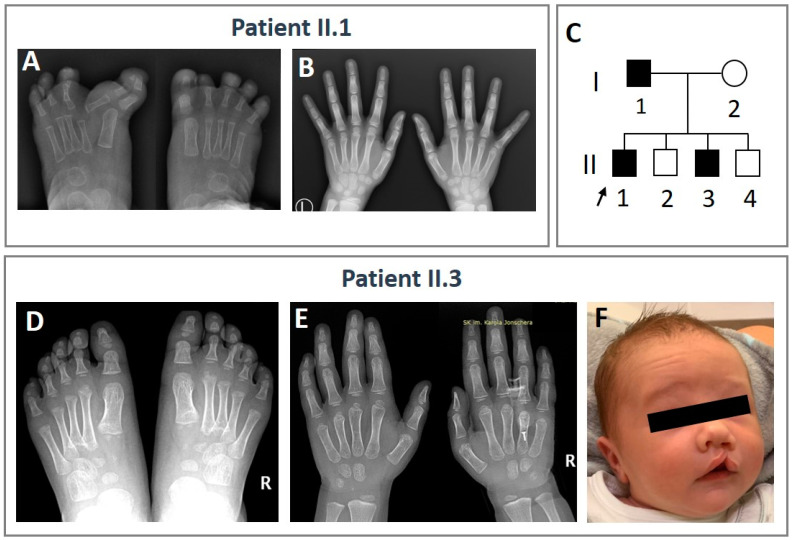
Clinical features observed in members of the affected family. (**A**) Spectrum of ectrodactyly of the left foot and synpolydactyly of the right foot diagnosed in the proband (II.1). (**B**) X-ray image of the unaffected hands of the proband (II.1). (**C**) The pedigree of the described family. The index patient is indicated by an arrow. (**D**) Bilateral deformities of the second and third metatarsals. (**E**) Irregular ossification centers in the hands of the proband’s brother (II.3). (**F**) Craniofacial malformations observed in the proband’s brother.

We report a Polish family comprising four siblings born to non-consanguineous parents. Clinical data of the family are summarized in Figure 1. The proband (II.1), a 7-year-old male, was born to healthy parents following an uneventful first pregnancy, except for a maternal viral infection around the 11th week of gestation. Delivery occurred at 39 weeks via cesarean section. At birth, the proband’s weight was 4110 g (97th percentile), with a height of 59 cm (97th percentile), head circumference of 37 cm (97th percentile), and Apgar score of 10. He presented with ectrodactyly of the left foot (cleft foot and polydactyly of the third toe) and synpolydactyly of the right foot (first and second toe syndactyly and polydactyly of the third toe). The hands were unaffected. Psychomotor development was mildly delayed, and autism spectrum disorder was diagnosed in early childhood.

The proband’s 4-year-old brother (II.3) exhibited bilateral deformities of the second and third metatarsals; irregular ossification centers in the hands; and craniofacial malformations, including a CL with alveolar ridge clefting, high forehead, narrow palpebral fissures, micrognathia, and low-set ears. He also had a history of recurrent viral infections. The third sibling (II.2), a 5-year-old boy, was diagnosed with ADHD (attention-deficit/hyperactivity disorder). The youngest brother (II.4), aged 1 year, also experienced recurrent infections. The proband’s father (I.1) exhibited hyposmia. Hormonal assessment, including follicle-stimulating hormone (FSH), luteinizing hormone (LH), testosterone, and estradiol (E2), revealed values within normal ranges. The mother (I.2) was phenotypically asymptomatic. There was no reported history of SHFM or CL and intellectual disability among close relatives on either side.

### 3.2. Genetic Analyses

**Figure 2:**
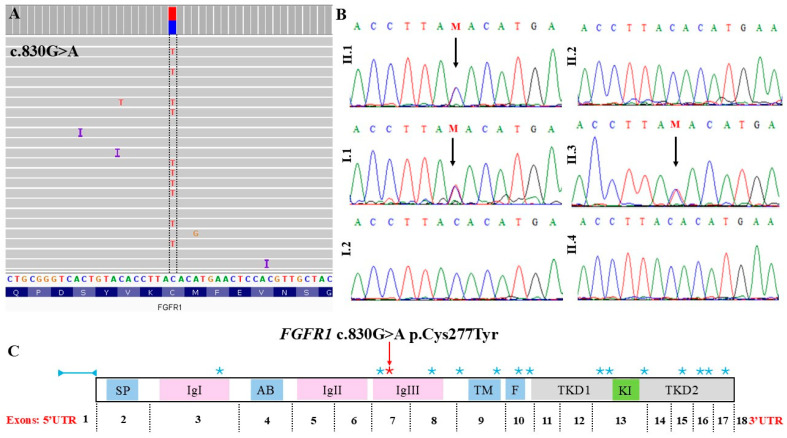
Molecular results of the studied family members. (**A**) Targeted next-generation sequencing results showing the heterozygous pathogenic variant c.830G>A p.(Cys277Tyr) within the *FGFR1* gene in the proband (II.1). The pathogenic single-nucleotide variant was visualized using Integrative Genomics Viewer (IGV). The reads were mapped to the reverse strand. (**B**) Sanger sequencing results of the analyzed family showing the pathogenic variant in affected family members (I.1, II.1, and II.3) and its absence in healthy individuals (I.2, II.2, and II.4). (**C**) Schematic representation of *FGFR1* showing the positions of *FGFR1* variants identified in the analyzed family (red asterisk) and previously reported cases with phenotypic overlap (blue asterisks). Deletion reported by Papasozomenou et al. (2019) [15] is indicated by a blue double-arrowhead symbol. SP—signal peptide; IgI, IgII, IgIII—Ig-like domains; AB—acidic box; TM—transmembrane domain; F—FRS2α-binding domain; KI—kinase insert; TKD—tyrosine kinase domain.

Targeted NGS for 25 SHFM-associated genes and SHFM-candidate genes (Table S1) revealed a heterozygous missense variant in *FGFR1* (NM_023110.3: c.830G>A; p.(Cys277Tyr)) in the proband. Sanger sequencing confirmed this variant in the patient, his father, and his brother affected by CL. However, the variant was absent in the proband’s mother and two siblings without congenital structural malformations. Figure 2 presents the results of molecular screening and segregation analyses. This variant has not been previously reported in population and clinical databases (gnomAD and ClinVar). It was predicted to be disease-causing by MutationTaster, CADD, SIFT, and REVEL (Table 1). According to the ACMG guidelines, the identified variant is classified as pathogenic (ACMG criteria: PP3 strong, PM1 moderate, PP5 moderate, PM2 supporting, and PP1 supporting).

## 4. Discussion

In this study, we report a familial case in which one individual presented with SHFM, the other one with CL and craniofacial dysmorphism, and the third showed hyposmia. In all of the affected individuals, the symptoms were most probably caused by a missense pathogenic variant in the *FGFR1* gene (c.830G>A; p.Cys277Tyr) that resulted in variable manifestation of the disorder in the affected family members. The index patient was diagnosed with a spectrum of feet ectrodactyly, autism, and mild developmental delay. In contrast, his brother presented with CL and minor digital anomalies but did not manifest developmental delay. Interestingly, the father (I.1), who carries the same variant, exhibited hyposmia without limb deformities. None of the affected family members exhibited CHH; therefore, they could not have been diagnosed as having isolated CHH or KS. The mother and two unaffected brothers did not carry the variant and were unaffected. Table 2 summarizes previously described pathogenic *FGFR1* variants associated with the phenotypes observed in our family. Additionally, Figure 2C illustrates the location of these variants within the *FGFR1* gene. Integration of data from Table 2 and Figure 2C indicates that patients with pathogenic variants affecting the same *FGFR1* domains shared similar clinical manifestations. The following discussion compares our findings with previous reports, refines genotype–phenotype correlations, and expands the known spectrum of *FGFR1*-related disorders.

FGFR1 is an important signaling molecule involved in a wide range of developmental processes, including the formation of skeletal, craniofacial, and olfactory structures. The role of FGFR1 in limb development has been established in studies using conditional *Fgfr1* mice knockouts, which exhibited aplasia of digits in the central and anterior rays of the autopods, along with syndactyly of digits III and IV [19]. This observation explains limb malformation in patients affected by KS, congenital hypogonadotropic hypogonadism, and Hartsfield syndrome [16,17,23]. In addition to the syndromic forms of limb anomalies, including ectrodactyly, caused by *FGFR1* mutations, isolated SHFM has been observed in a single family harboring a c.787_789del (p.Ala263del) heterozygous variant in the *FGFR1* gene [15]. To the best of our knowledge, the index patient described in our study is the second case affected by isolated SHFM resulting from the *FGFR1* loss-of-function variant [15].

Fibroblast growth factor (FGF) is a ligand of FGFR1, which is a tyrosine kinase receptor, and when it is bound to FGFR1, the binding (activation) sends important signaling for a variety of cellular processes, such as proliferation, differentiation, migration, and apoptosis. It plays a role in normal embryonic development and bone and other tissue formation, especially in the development of craniofacial bones and skeletal bones in the arms, hands, legs, and feet. Beyond the involvement of FGFR1 in the development of skeletal features, the protein plays a crucial role in the formation and differentiation of the olfactory system, where it is required for the development of olfactory sensory neurons and fate specification of gonadotropin-releasing hormone (GnRH) neurons. FGFR1 stimulates the proliferation and migration of GnRH neurons from the olfactory placode to the hypothalamic region [24]. Thus, the loss-of-function mutations in *FGFR1* cause failed morphogenesis of the olfactory bulbs, leading to anosmia or hyposmia and CHH observed in patients with KS [8]. We observed isolated hyposmia in the proband’s father, who carries the *FGFR1* variant. A comprehensive hormonal assessment ruled out CHH at the time of evaluation, suggesting that KS is unlikely but cannot be entirely excluded.

According to medical literature, this case represents the second report of isolated hyposmia associated with a pathogenic variant in *FGFR1*. Previously, researchers documented congenital hyposmia in the mother of a patient with partial normosmic idiopathic hypogonadotropic hypogonadism, unilateral cryptorchidism, and two congenitally missing teeth. The patient and his mother harbored a heterozygous double pathogenic variant in the *FGFR1* gene (p.Pro722His and p.Asn724Lys) [25]. These findings refine the phenotypic spectrum of *FGFR1* variants, indicating that isolated hyposmia can manifest independently rather than in association with KS or CHH.

Expanding the phenotypic scope observed within the family, we identified craniofacial anomalies in the proband’s brother (II.3). The FGF signaling pathway regulates proper palatogenesis and craniofacial morphogenesis. Mice studies have demonstrated that *Fgfr1* deletion in cranial neural crest cells disrupts cellular signaling and reduces cell proliferation. These defects impaired palatal shelf elevation and fusion, leading to abnormal palatogenesis. Phenotypically, *Fgfr1*-deficient mice exhibited CP, CL, micrognathia, a heightened tongue, and other craniofacial malformations [26]. These observations align with our findings and suggest the potential involvement of variants in *FGFR1* in the craniofacial anomalies identified in the patient (II.3). Table 2 shows that CL can occur as an isolated anomaly [27] or as a syndromic constituent in patients with pathogenic variants in *FGFR1* [16,17]. CL/P has previously been associated with KS or SHFM in association with CHH. We report the first patient with CP associated with SHFM with no current clinical or biochemical evidence of KS or CHH. However, given the known variable expressivity and incomplete penetrance associated with *FGFR1* pathogenic variants, early manifestations of KS or CHH—such as absent or delayed puberty—may still emerge during adolescence. This highlights the importance of long-term clinical follow-up in patients with pathogenic variants in *FGFR1*, even without early signs of hypogonadism.

From a genetic perspective, the variant identified in the family (p.Cys277Tyr) affects a highly conserved cysteine residue within the third Ig-like domain of FGFR1 (Figure 2C). This residue is one of two cysteines essential for forming a disulfide bond that maintains the structural integrity and functionality of FGFR1. Disruption of this bond may alter receptor conformation and impair downstream signaling. This variant has been previously reported in two patients with KS; however, their clinical manifestations differed from those observed in our patients [9,28].

The variable expressivity of *FGFR1*-related disorders, where the same mutation can lead to different clinical outcomes in individuals within or between affected families, may result from a number of phenomena, such as the pleiotropy of *FGFR1* function, modifier genes, environmental factors, epigenetic modifications, or regulatory variations that affect gene expression. To date, several modifier genes have been identified to influence the phenotypic expression of *FGFR1* mutations, particularly in KS and CHH. According to the recent findings, approximately 10% of CHH/KS patients carry mutations in more than one gene, suggesting a digenic or oligogenic mode of inheritance [29,30]. The identified *FGFR1* gene partners include *GNRHR*, *NSMF* (*NELF*), *FGF8*, and *SEMA3A*, in which co-occurring mutations contribute to more severe phenotypes, suggesting a synergistic effect of variants in the given gene pair [30,31,32,33]. All of the above-listed genes mediate the action of GnRH neurons and were previously described in association with CHH, normosmic IHH, or KS [31,32,33]. These findings support the concept of oligogenic inheritance of congenital disorders characterized by variable expression or incomplete penetrance in which the phenotypic spectrum depends on the co-occurrence of certain variants in disease-associated genes.

In conclusion, our findings expand the phenotypic spectrum associated with pathogenic variants in *FGFR1*. This study reveals that pathogenic variants in *FGFR1* can cause isolated SHFM, highlighting the need for further research into its involvement in the disease. Additionally, we demonstrate the clinical utility of targeted NGS panels as a cost-effective approach for the molecular analysis of patients presenting with SHFM. While this approach has limitations in detecting copy number variations, variants located in untranslated regions, and exon–intron boundaries, the method can still yield diagnostic information. Specific genetic findings may provide patients with accurate genetic counseling and help develop a strategy for managing future pregnancies.

**Table 2:** Reported cases with genetic and phenotypic overlap with our family caused by pathogenic variants in *FGFR1*. Abbreviations: *—presence of symptoms in family members, CHH—congenital hypogonadotropic hypogonadism, CLP—cleft lip and palate, F—female, KS—Kallmann syndrome, M—male, N.E.—not examined, SHFM—split/hand foot malformation. Note: annotation reference sequence NM_023110.3 (ENST00000447712).

Variant	Gender	Inheritance	Diagnosis	SHFM	CL/P	Isolated Anosmia/Hyposmia	References
NC_000008.10:g.38320713_38329024del	M	Maternal	SHFM + CHH	Both hands, Both feet	Yes	No	[16]
c.289G>A p.(Gly97Ser)	M	N.E.	SHFM + CHH	Right hand	No	No	[16]
c.787_789del p.(Ala263del)	M (fetus)	Paternal *	SHFM	Both hands, Right foot	No	No	[15]
**c.830G>A p.(Cys277Tyr)**	M (II.1)	Paternal *	SHFM	**Both feet**	No	No	**Our report**
	M (II.3)		CL, minor digital anomalies	No	**Yes**	No	
	M (I.1)		Isolated hyposmia	No	No	**Yes**	
	F	Maternal	KS	No	No	No	[9]
	M	N.E.	KS	No	No	No	[28]
c.1042G>A p.(Gly348Arg)	M	N.E.	CHH	Both feet	Yes	No	[17]
c.1107G>A p.(Met369Ile)	F	Maternal *	CLP	No	Yes	No	[27]
c.1286T>A p.(Val429Glu)	M	N.E. *	KS	Both hands, Both feet	No	No	[17]
c.1399G>A p.(Glu467Lys)	M	N.E.	CLP	No	Yes	No	[27]
c.1435G>A p.(Gly485Arg)	M	Maternal *	CHH	Right foot	Yes	No	[17]
c.1663+1G>T	F	N.E.	CHH	Left hand	No	No	[16]
c.1780C>T p.(Gln594Ter)	M	N.E.	CHH	Both feet	No	No	[17]
c.1825C>T p.(Arg609Ter)	F	Paternal *	CLP	No	Yes	Yes	[27]
c.2009G>A p.(Glu670Ala)	M	N.E. *	CHH	Both feet	Yes	No	[17]
c.2062G>T p.(Val688Leu)	M	Maternal *	KS	Left foot	Yes	No	[17]
c.2135T>C p.(Leu712Pro)	M	N.E.	KS	Both feet	No	No	[17]
c.2231G>C p.(Arg744Thr)	M	N.E.	CHH + SHFM	Both hands, Both feet	No	No	[16]

## Figures and Tables

**Table 1:** Summary of the pathogenic variant reported in our paper.

Gene	*FGFR1*
Genomic position (hg19)	Chr8:38282133
Reference sequence number	NM_023110.3
Nucleotide change	c.830G>A
Protein change	p.Cys277Tyr
Inheritance	Paternal
Location	Exon 7
ACMG Classification	Pathogenic (PP3 strong, PM1 moderate, PP5 moderate, PM2 supporting, and PP1 supporting)
CADD	33
SIFT	0
REVEL	0.962
MutationTaster	Disease-causing
PolyPhen	1.0

## Data Availability

Data generated during this study are included in this published article and its Supplementary Materials.

## Supplementary Materials

The following supporting information can be downloaded at: https://www.mdpi.com/article/10.3390/genes16050495/s1, Table S1: A list of the genes included in the targeted next-generation sequencing panel. Table S2: The oligonucleotide primers used to perform PCR and Sanger sequencing.
