# Supplementary material for: Intrafamilial Phenotypic Variability of the FGFR1 p.Cys277Tyr Variant: A Case Report and Review of the Literature
Source: Genes (Basel). 2025 Apr 26;16(5):495. doi: 10.3390/genes16050495 (PMC12110769; doi:10.3390/genes16050495)
Supplement: Supplementary file 1 [file genes-16-00495-s001.zip › Supplementary Table S2.pdf]

Table S2 Oligonucleotide primers used to perform PCR and Sanger sequencing

Primer name	sequence (5'->3')	Genomic coordinates (hg38)	Target
FGFR1_F	AATGCCTTCCTTGTGTAGCT	chr8:38424212-38424818	*FGFR1*
FGFR1_R	AAGTGCTGGGAGGTTTACAA		
